# Typing Style and the Use of Different Sources of Information during Typing: An Investigation Using Self-Reports

**DOI:** 10.3389/fpsyg.2016.01908

**Published:** 2016-12-09

**Authors:** Martina Rieger, Victoria K. E. Bart

**Affiliations:** Department for Psychology, Medical Sciences and Health Systems Management, Institute for Psychology, University for Health Sciences, Medical Informatics and TechnologyHall in Tirol, Austria

**Keywords:** typing style, attention to sources of information, copy typing, free typing, error detection

## Abstract

We investigated to what extent different sources of information are used in typing on a computer keyboard. Using self-reports 10 finger typists and idiosyncratic typists estimated how much attention they pay to different sources of information during copy typing and free typing and how much they use them for error detection. 10 finger typists reported less attention to the keyboard and the fingers and more attention to the template and the screen than idiosyncratic typists. The groups did not differ in attention to touch/kinaesthesis in copy typing and free typing, but 10 finger typists reported more use of touch/kinaesthesis in error detection. This indicates that processing of tactile/kinaesthetic information may occur largely outside conscious control, as long as no errors occur. 10 finger typists reported more use of internal prediction of movement consequences for error detection than idiosyncratic typists, reflecting more precise internal models. Further in copy typing compared to free typing attention to the template is required, thus leaving less attentional capacity for other sources of information. Correlations showed that higher skilled typists, regardless of typing style, rely more on sources of information which are usually associated with 10 finger typing. One limitation of the study is that only self-reports were used. We conclude that typing task, typing proficiency, and typing style influence how attention is distributed during typing.

## Introduction

Due to the widespread use of computers typing is a frequent activity in everyday life for many people. Typing is performed using different techniques, from the use of one finger of each hand (“hunt and peck typing”) to the use of all 10 fingers (also referred to as touch typing, indicating that it is usually performed without looking at the keyboard; Cooper, 1983; West, 1983). So far, only little is known about how typing-related processes, representations, and the distribution of attention differ in 10 finger typists and idiosyncratic typists, because typists of different styles are rarely compared (but see Long et al., 1983; Jordan, 1995; Rieger, 2004, 2007, 2012; Beilock and Holt, 2007). Further, most studies are conducted on copy typing, some on error detection, but free typing is rarely investigated. In the present study we therefore investigated the role of different typing styles for the distribution of attention to different sources of information. We also took skill level within typing styles into account. Using self-reports, 10 finger typists and idiosyncratic typists estimated the extent of attention to different sources of information (template, screen, keyboard, fingers, and touch/kinaesthesis) during copy typing and free typing. We further investigated which of these sources of information (including internal prediction as an additional source of information) are used for error detection.

The importance of different sources of information in typing is emphasized in contemporary models of typewriting, such as the two-loop theory of typewriting (Logan and Crump, 2009). This theory proposes a hierarchical control system consisting of two nested feedback loops (outer loop and inner loop). The outer loop transforms text and thoughts into a series of words and the inner loop transforms words provided by the outer loop into a series of keystrokes (Logan and Crump, 2009, 2010; Liu et al., 2010). The two loops rely on different kinds of feedback: The inner loop is responsible for generating keystrokes, monitoring the fingers, and the keyboard, and processes tactile and kinaesthetic feedback in order to ensure that a typist moves the right fingers and hits the correct keys (Liu et al., 2010; Logan and Crump, 2011). The outer loop monitors the screen to ensure that the intended word matches the actual one. In case of a mismatch, the inner loop has to correct the error (Logan and Crump, 2011).

During typing *movement related information* is provided by the fingers and their movement into the right direction and the keyboard to find and hit the correct key. Feedback about the movement (*feedback related information*) is provided by letters appearing on the computer screen, tactile feedback (the sensation of hitting the keys), and kinaesthetic feedback (the sensation of feeling the movement of the fingers). During copy typing, the template is an additional source of information. For error detection another source of information may be predictive processes, which predict movement consequences based on current motor commands (Rabbitt, 1978; Wolpert and Flanagan, 2001; Maidhof et al., 2009; see below). In the following we discuss each of these sources of information and how attention to them might be influenced by typing style.

The template is an obvious source of information in copy typing regardless of the typing style as it is necessary for typing a copy of the text. Thus, attention to it is non-optional. When 10 finger typists perceive letters, the corresponding finger used to type the key (effector dependent representation) and the spatial representation of the location of the key are automatically activated (Van den Bergh et al., 1990; Rieger, 2004; Beilock and Holt, 2007). Thus, for 10 finger typists it may not be necessary to devote a lot of attentional resources to the actual movements, and they may therefore devote a lot of attention to the template. However, idiosyncratic typists may frequently need to look away from the template. They do not show such automatic activation (Rieger, 2004; Beilock and Holt, 2007) and their typing style may require them to visually search for the right keys on the keyboard.

Visual feedback consisting of text appearing on the screen enables a typist to monitor, whether she or he actually produced the desired output. In particular, the screen plays an important role for error correction by providing information about the type of error that has occurred (West, 1967; Long, 1976). Continuous attention to the screen is one key characteristic of 10 finger typing (as learned in touch typing lessons), even though not all typists may adhere to this strict rule. Correspondingly, results concerning the screens' importance are contradictory. Diehl and Seibel (1962) found that covering the sheet of paper in a typewriter does not reduce typing speed or accuracy. However, skilled typists do take frequent looks at the screen in order to read the text they have typed (Johansson et al., 2010). Therefore, 10 finger typists may pay more attention to the screen than idiosyncratic typists, because idiosyncratic typing often requires looking at the keyboard to search for the right keys, which may result in less attention to the screen.

Movement related information from (a) the fingers, i.e., the fingers' movement into the right direction, and (b) corresponding information about the location of keys on the keyboard, seems to be particularly important for idiosyncratic typists in order to find and hit the correct keys. Movement related information should be less important for 10 finger typists (see above). Still, 10 finger typists might use information from the keyboard and fingers at least to some degree. Covering the fingers and the keyboard leads to decreasing typing speed and accuracy in 10 finger typists (Hayes and Reeve, 1980; Tapp and Logan, 2011). This might be due to the importance of information from the keyboard to find rarely used keys (Long, 1976). Thus, we expect that 10 finger typist do occasionally use information from the keyboard and fingers during typing, but should do less so than idiosyncratic typists.

Tactile (touching the keys and feeling the pressure of hitting them) and kinaesthetic (feeling of the movements of the fingers) feedback enables typists to detect and correct about 60–70% of typing errors when the keyboard and the copy in the typewriter are covered (Rabbitt, 1978). Loss of tactile feedback (Gordon and Soechting, 1995; Rabin and Gordon, 2004) or only little tactile feedback (Roeber et al., 2003; Crump and Logan, 2010) leads to more errors, problems with detecting and correcting errors, and slower typing speed in 10 finger typists. Further, 10 finger typists use tactile/kinaesthetic feedback to determine the key locations on the keyboard (Liu et al., 2010). Tactile/kinaesthetic feedback seems to be less important in idiosyncratic typists, as typing on keyboards with different or little tactile feedback (i.e., piezoelectric or membrane keyboards), leads to less impairment of typing performance (speed and accuracy) in idiosyncratic typists than in 10 finger typists (Loeb, 1983; Barrett and Krueger, 1994). We therefore expect that 10 finger typists pay more attention to touch/kinaesthesis than idiosyncratic typists.

Internal prediction refers to the idea that when a movement is performed, movement consequences (such as vision and kinaesthesis) are predicted based on an efference copy of the motor command (comparator model, Wolpert and Flanagan, 2001). According to the comparator model of motor control errors can be detected due to comparisons of three different signals about movement consequences: intended movement consequences, observed movement consequences, and internally predicted movement consequences. The comparison of intended movement consequences and observed movement consequences and the comparison of predicted movement consequences and observed movement consequences are only possible if actual feedback (here: vision and kinaesthesis) is available. However, the comparison of intended movement consequences and internally predicted movement consequences can lead to the detection of (some) errors in the absence of feedback. Errors may be detected even before the action is fully executed (i.e., before the actual movement consequence is available), even though they may still be committed (Maidhof et al., 2009). Subjectively, internally prediction may result in the impression that something is about to go wrong in the absence of any other feedback. It is likely that such predictive processes take place during typing (Rabbitt, 1978; Rieger et al., 2011). For instance, skilled typists execute an erroneous keystroke with less force than a correct one. This indicates that typists may (consciously or unconsciously) realize that they are about to commit an error before they complete the keystroke and they may try to inhibit the keystroke (Rabbitt, 1978). One may assume that internal models of typing are more finely tuned in 10 finger typists than idiosyncratic typists, because motor commands are more specific for letters in 10 finger typists. Thus, to detect errors 10 finger typists may use internal prediction more often than idiosyncratic typists.

In sum, the relative importance of different sources of information within 10 finger typists and idiosyncratic typists may differ because the two typing styles differ in the assignment of fingers to keys. Due to higher automaticity of movements (cf. Rieger, 2004; Beilock and Holt, 2007), 10 finger typists may pay more attention to feedback related information, that is, touch/kinaesthesis and visual feedback on the screen, and less attention to movement related information, that is, the fingers and the keyboard. In contrast, idiosyncratic typists may pay more attention to movement related information, which diminishes attentional resources for feedback related information. This should be the case for copy typing, free typing, and error detection.

Attention to different sources of information may not only depend on typing style, but also on typing task. In copy typing it is necessary to attend to the template, which does not exist in free typing. Thus, we predict that attention to visual feedback on the screen and movement related information from the fingers and the keyboard should be lower in copy typing than in free typing, because they cannot be attended to at the same time. However, we expect that attention to touch/kinaesthesis does not differ between free typing and copy typing, because it should be possible to attend to touch/kinaesthesis and the template simultaneously. These effects may be influenced by typing style. Because we assume that 10 finger typists do not pay much attention to movement related sources of information, we expect that attention to movement related information does not differ between copy typing and free typing in 10 finger typists. In addition, we do not expect that attention to the screen differs between free typing and copy typing in idiosyncratic typists, because we assume that idiosyncratic typists do not attend to the screen very often.

Because of limited attentional and perceptual resources some sources of information should be prioritized at the cost of others. This should be the case within both groups of typists. We assume that positive correlations should be observed between attention to sources of information to which attention can be paid at the same time (e.g., the screen and touch/kinaesthesis) and that negative correlations should be observed between sources of information to which it is difficult to pay attention at the same time (e.g., the screen and the fingers).

Typing ability varies widely, and some idiosyncratic typists may be proficient in their style, because they may use it on an everyday basis. Higher skilled typists of each style should have more finely tuned internal models for typing. We therefore assume that within each group of typists, higher skilled participants pay more attention to feedback related information and less attention to movement related information.

Not all idiosyncratic typists type the same way. Most obviously, they use different number of fingers. The use of more fingers may result in a way of distributing attention, which is similar to typing using 10 fingers. We expect that idiosyncratic typists who use more fingers use feedback related information more and movement related information less than typists who use less fingers.

## Methods

### Participants

Originally 298 participants took part in the study. Because of missing data five participants had to be excluded from analysis. The remaining participants were assigned to two groups depending on their typing style: typing using 10 fingers (10 finger typists) and typing using an idiosyncratic style (idiosyncratic typists). In Table 1 demographic data and data related to typing ability and typing experience of the two groups are depicted. *T*-tests indicate significantly/a trend to higher performance and more experience in 10 finger typists than idiosyncratic typists. The study was approved by the local ethics committee and all participants gave informed consent. The whole study took ~15 min.

**Table 1:** **Demographic data**.

	**10 finger typists**	**Idiosyncratic typists**	**df**	***t***	***p***
	***N* = 132**	***N* = 161**			
Sex (female/male)	98/34	94/67	–	–	–
Education (university degree/higher secondary school qualification/lower secondary school qualification)	12.2/61.6/26.7%	20.6/65.3/23.1%	–	–	–
Age *M*(*SD*)	31.3 (12.6)	31.2 (12.8)	291	0.041	0.97
Self-reported typing ability *M*(*SD*)	68.6 (20)	43 (23)	291	10.3	<0.001
Years using a keyboard *M*(*SD*)	17.9 (10.5)	15.7 (9)	289	1.95	0.05
Hours typing/week *M*(*SD*)	13.2 (12.1)	10.6 (11.3)	288	1.93	0.06
No. of fingers used for typing *M*(*SD*)	–	6.58 (2.52)	–	–	–

*Means and standard deviations of typing ability and typing experience separately for 10 finger typists and idiosyncratic typists, and results of t-tests for group comparisons*.

### Material, procedure, and design

All participants were asked to complete a questionnaire starting with demographic data and questions about typing ability (see Supplemental Material, Section A). Participants were also asked to specify which fingers they use to type and to state their typing style (10 finger typing style, idiosyncratic style). Afterwards they were asked 14 questions to indicate to which extent they pay attention to different sources of information during free typing and copy typing, and which sources of information they use to detect errors (see Supplemental Material, Section B). The questions referred to looking at the keyboard, looking at the fingers, looking at the screen, and the feeling of moving the fingers (touch/kinaesthesis). In addition, for copy typing a question concerning looking at the template and for error detection a question concerning internal prediction was added. To assess internal prediction, participants were asked about “detecting a typing error even if they do not look at the screen, the keyboard, or the fingers and their finger movements do not feel weird. They just know without this information that something is about to go wrong.” The questions were answered on a five-point Likert scale (never, rarely, sometimes, often, and always). In order to assess typing ability, participants rated their ability on a 100 millimetre visual analogue scale (from 1 = very bad to 100 = very good).

### Data analysis

The data on attention to/use of different sources of information for free typing, copy typing, and error detection were analyzed using analysis of variances (ANOVAs). For all effects with variables with more than two levels we report Greenhouse-Geisser corrected *F*-values and *p*-values, and Greenhouse-Geisser'ε. *Post-hoc* comparisons were conducted using additional ANOVAs and *t*-tests. The significance level for *post-hoc* tests was corrected using the Holm-Šídák procedure. Where appropriate exact, minimum (*p*_min_), and/or maximum (*p*_max_) *p*-values are reported. Pearson product moment correlations were computed in order to investigate (a) correlations between attention to the different sources of information with each other, (b) correlations between attention to the different sources of information and typing skill, and (c) correlations between attention to the different sources of information and the number of fingers in idiosyncratic typists. Correlations were compared using Fisher's *z*-test.

## Results

### Sources of information during copy typing

**Figure 1:**
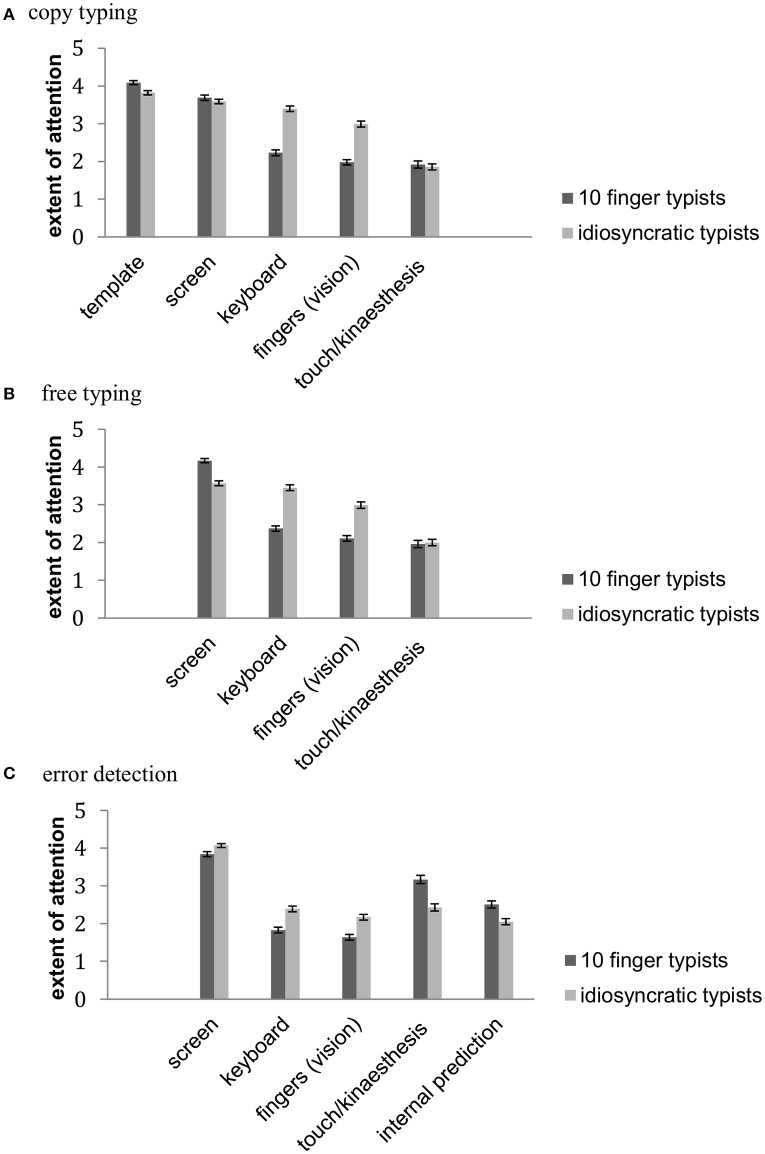
**Means and standard errors of the reported extent of attention to different sources of information in (A)** copy typing, **(B)** free typing, and **(C)** the use of the sources of information for error detection in 10 finger typists and idiosyncratic typists.

Means and standard errors of attention to different sources of information during copy typing can be seen in Figure 1A. An ANOVA with the factors typing style (idiosyncratic typing, 10 finger typing) and sources of information (template, fingers, keyboard, screen, and touch/kinaesthesis) was computed on the reported extent of attention. The results of the ANOVA can be seen in Table 2. The significant main effects of typing style and sources of information were modified by a significant interaction between typing style and sources of information. *Post-hoc* comparisons showed that 10 finger typists reported more attention to the template (*p* = 0.001) and less to the fingers (*p* < 0.001) and the keyboard *(p* < 0.001) than idiosyncratic typists. No significant differences between the groups were observed in attention to the screen (*p* = 0.27) and attention to touch/kinaesthesis (*p* = 0.63). 10 finger typists reported least attention to touch/kinaesthesis, the fingers and the keyboard, which did not significantly differ from each other, apart from that they reported more attention to the keyboard than to the fingers (*p* = 0.016, all other comparisons p_min_ = 0.14). They reported more attention to the screen (*p*_max_ < 0.001), and even more to the template (*p*_max_ < 0.001). Idiosyncratic typists reported least attention to touch/kinaesthesis (*p*_max_ < 0.001), followed by attention to the fingers (*p*_max_ < 0.001), the keyboard, and the screen. In contrast to the results in 10 finger typists, attention to the latter two sources of information did not significantly differ from each other (*p* = 0.35). They reported most attention to the template (*p*_max_ = 0.009).

**Table 2:** **Results of the ANOVAs**.

	**df, ε**	***F***	***p***	**$\eta_{p}^{2}$**
**COPY TYPING**				
Typing style	1, 291	48.4	<0.001	0.14
Sources of information	4, 1164, ε = 0.09	274	<0.001	0.49
Typing style ^*^ sources of information	4, 1164, ε = 0.09	45	<0.001	0.13
**FREE TYPING**				
Typing style	1, 291	38.1	<0.001	0.12
Sources of information	3, 873, ε = 0.91	203	<0.001	0.42
Typing style ^*^ sources of information	3, 873, ε = 0.91	49	<0.001	0.14
**COPY TYPING VS. FREE TYPING**				
Typing style	1, 291	58.9	<0.001	0.97
Typing task	1, 291	32.1	<0.001	0.099
Sources of information	3, 873, ε = 0.9	232	<0.001	0.45
Typing style ^*^ typing task	1, 291	13.3	<0.001	0.044
Typing style ^*^ sources of information	3, 873, ε = 0.9	52	<0.001	0.15
Typing task ^*^ sources of information	3, 873, ε = 0.93	3.67	0.014	0.012
Typing task ^*^ typing style ^*^ sources of information	3, 873, ε = 0.93	9.75	<0.001	0.032
**ERROR DETECTION**				
Typing style	1, 291	0.24	0.62	0.001
Sources of information	4, 1164, ε = 0.85	217	<0.001	0.43
Typing style ^*^ sources of information	4, 1164, ε = 0.85	28.4	<0.001	0.089

*The first two ANOVAs were performed with the factors typing style and sources of information on the reported extent of attention in copy typing and in free typing. The third ANOVA with the factors typing style, typing task, and sources of information was performed on the reported extent of attention. The fourth ANOVA with the factors typing style and sources of information was performed on the use of cues in error detection*.

### Sources of information during free typing

Means and standard errors of attention to different sources of information during free typing can be seen in Figure 1B. An ANOVA with the factors typing style (idiosyncratic typing, 10 finger typing) and sources of information (fingers, keyboard, screen, and touch/kinaesthesis) was computed on the reported extent of attention. The results of the ANOVA can be seen in Table 2. The significant main effects of typing style and sources of information were modified by a significant interaction between typing style and sources of information. The interaction indicated that 10 finger typists pay more attention to the screen (*p* < 0.001) and less attention to the fingers *(p* < 0.001) and to the keyboard (*p* < 0.001) than idiosyncratic typists. No significant difference between the groups was observed in attention to touch/kinaesthesis (*p* = 0.77). 10 finger typists reported least attention to touch/kinaesthesis and to the fingers. These did not significantly differ from each other (*p* = 0.78). More attention was reported to the keyboard (*p*_max_ = 0.005) and even more to the screen (*p*_max_ < 0.001). Idiosyncratic typists, reported least attention to touch/kinaesthesis (*p*_max_ < 0.001), more attention to the fingers (*p*_max_ < 0.001), and most attention to the keyboard and the screen (*p*_max_ < 0.001). Attention to the latter two sources of information did not significantly differ (*p* = 0.88).

### Comparison of copy typing and free typing

An ANOVA with the factors typing style (idiosyncratic typing, 10 finger typing), typing task (copy typing, free typing), and sources of information (touch/kinaesthesis, fingers, keyboard, and screen) was computed on the reported extent of attention. The results of the ANOVA can be seen in Table 2. All main effects and two-way interactions were modified by a significant interaction between typing style, typing task, and sources of information. To explain this interaction, we computed the interaction between typing task and sources of information separately for 10 finger typists and idiosyncratic typists. In 10 finger typists the interaction was significant, *F*_(3, 393)_ = 11.56, *p* < 0.001, $\eta_{p}^{2}$ = 0.081, ε = 0.94, indicating that 10 finger typists reported more attention to the keyboard (*M*_diff_ = 0.14, *p* = 0.007) and the screen (*M*_diff_ = 0.49, *p* < 0.001) in free typing than in copy typing. No significant differences between free typing and copy typing were observed in attention to touch/kinaesthesis (*M*_diff_ = 0.038, *p* = 0.44) and the fingers (*M*_diff_ = 0.12, *p* = 0.052). In idiosyncratic typists the interaction between typing task and sources of information was not significant, *F*_(3, 480)_ = 1.6, *p* = 0.19, $\eta_{\text{p}}^{2}=$ 0.01, ε = 0.91, indicating that attention to different sources of information did not differ between free typing and copy typing.

### Sources of information during error detection

Means and standard errors of the use of different sources of information during error detection can be seen in Figure 1C. An ANOVA with the factors typing style (idiosyncratic typing, 10 finger typing) and sources of information (fingers, keyboard, screen, touch/kinaesthesis, and internal prediction) was computed on the reported use for error detection. The results of the ANOVA can be seen in Table 2. There was no significant main effect of typing style, but a significant main effect of sources of information, which was modified by a significant interaction between typing style and sources of information. The interaction indicated that 10 finger typists reported more use of touch/kinaesthesis (*p* < 0.001) and internal prediction (*p* < 0.001) and less use of the fingers *(p* < 0.001), the keyboard (*p* < 0.001), and the screen (*p* = 0.008) than idiosyncratic typists. 10 finger typists reported to use the fingers and the keyboard (which did not significantly differ from each other, *p* = 0.06) less than all other sources of information (*p*_max_ < 0.001). They reported more use of internal prediction (*p*_max_ < 0.001), even more of touch/kinaesthesis (*p*_max_ < 0.001), and yet more of the screen (*p*_max_ < 0.001). In idiosyncratic typists use of fingers did not significantly differ from use of internal prediction, the keyboard, and touch/kinaesthesis (*p*_min_ = 0.06). However, they reported significantly less use of internal prediction than of the keyboard and touch/kinaesthesis (*p*_max_ = 0.029). The latter two did not significantly differ from each other (*p* = 0.99). Further, they reported to detect most errors by using the screen (*p*_max_ < 0.001).

### Correlations between different sources of information

In Table 3, correlations between the extent of attention to/use of different sources of information are shown separately for copy typing, free typing, and error detection and separately for 10 finger typists and idiosyncratic typists. In copy typing, 10 finger typists who reported more attention to the template reported less attention to movement related sources of information (fingers and keyboard) and the screen. More attention to the fingers coincided with more attention to the keyboard. In copy typing in idiosyncratic typists more attention to the template coincided with more attention to the screen. In contrast to 10 finger typists, attention to the template was not significantly correlated with attention to the fingers and the keyboard. However, less attention to the fingers coincided with more attention to the screen. Even though attention to the fingers and the keyboard was positively correlated as in 10 finger typists, this correlation was significantly lower than in 10 finger typists (*p* = 0.02). Further, attention to the fingers was positively correlated with attention to touch/kinaesthesis.

**Table 3:** **Correlations between the extent of attention to/use of different sources of information in copy typing, free typing, and error detection separately for 10 finger typists and idiosyncratic typists**.

	**Template**	**Keyboard**	**Fingers**	**Touch/kinaesthesis**	**Internal prediction**
**COPY TYPING**					
**10 finger typists**					
Screen	−0.23^**^	0.09	0.06	0.05	–
Keyboard	−0.38^***^	–	0.49^***^	0.04	–
Fingers	−0.29^**^	–	–	0.16	–
Touch/kinaesthesis	0.009	–	–	–	–
**Idiosyncratic typists**					
Screen	0.28^***^	0.01	−0.18^*^	0.11	–
Keyboard	0.06	–	0.26^**^	−0.05	–
Fingers	−0.1	–	–	0.19^*^	–
Touch/kinaesthesis	−0.07	–	–	–	–
**FREE TYPING**					
**10 finger typists**					
Screen	–	−0.41^***^	−0.38^***^	−0.02	–
Keyboard	–	–	0.56^***^	−0.02	–
Fingers	–	–	–	0.09	–
**Idiosyncratic typists**					
Screen	–	−0.23^**^	−0.24^**^	−0.02	–
Keyboard	–	–	0.29^***^	−0.07	–
Fingers	–	–	–	0.2^*^	–
**ERROR DETECTION**					
**10 finger typists**					
Screen	–	−0.12	−0.02	−0.41^***^	−0.4^***^
Keyboard	–	–	0.59^***^	0.06	0.07
Fingers	–	–	–	0.003	0.003
Touch/kinaesthesis	–	–	–	–	0.18
**Idiosyncratic typists**					
Screen	–	−0.06	−0.29^***^	−0.22^**^	−0.18^*^
Keyboard	–	–	0.49^***^	0.18^*^	−0.032
Fingers	–	–	–	0.21^**^	0.06
Touch/kinaesthesis	–	–	–	–	0.43^***^

* p < 0.05;

** p < 0.01;

*** *p < 0.001*.

In free typing the data pattern was similar. Again, positive correlations between fingers and keyboard were observed in both groups (the correlation was again higher in 10 finger typists than in idiosyncratic typists, *p* = 0.004). Less attention to the fingers and the keyboard coincided with more attention to the screen in both groups. In idiosyncratic typists attention to the fingers was again positively correlated with attention to touch/kinaesthesis.

Concerning error detection, both in 10 finger typists and idiosyncratic typists, detection of errors based on information from the fingers correlated positively with detection of errors based on information from the keyboard. Higher use of information from the screen coincided with less use of touch/kinesthesis and internal prediction. Additionally, in idiosyncratic typist, error detection based on information from the fingers was negatively correlated with error detection based on information from the screen. Further, more error detection based on touch/kinaesthesis coincided with more error detection based on information from the fingers, the keyboard, and internal prediction.

### Correlations between typing ability and attention to different sources of information

In Table 4 correlations between self-reported typing ability and the extent of attention to/use of different sources of information in copy typing, free typing, and error detection can be seen separately for 10 finger typists and idiosyncratic typists. In copy typing higher typing ability coincided with less attention to the keyboard and the fingers. Further, in 10 finger typists higher typing ability significantly coincided with more attention the template. Attention to touch/kinaesthesis and attention to the screen were not significantly correlated with typing ability in both groups.

**Table 4:** **Correlations between attention to/use of different sources of information and self-reported typing ability separately for 10 finger typists and idiosyncratic typists**.

	**Self-reported typing ability**					
	**10 finger typists** ***N*** = **132**			**Idiosyncratic typists** ***N*** = **161**		
	**Copy typing**	**Free typing**	**Error detection**	**Copy typing**	**Free typing**	**Error detection**
Template	0.34^***^	–	–	0.09	–	–
Screen	−0.05	0.16	−0.07	0.08	0.25^**^	−0.03
Keyboard	−0.42^***^	−0.4^***^	−0.25^**^	−0.51^***^	−0.54^***^	−0.2
Fingers	−0.28^**^	−0.4^**^	−0.12	−0.24^**^	−0.36^***^	−0.04
Touch/kinaesthesis	−0.03	−0.03	0.11	−0.002	−0.01	0.23^**^
Internal prediction	–	–	−0.02	–	–	0.18^*^

* *p < 0.05*,

** *p < 0.01*,

*** *p < 0.001*.

In free typing similar correlations were observed. Higher typing ability again coincided with less attention to the keyboard and with less attention to the fingers. Additionally, higher typing ability coincided with more attention to the screen in idiosyncratic typists. Attention to touch/kinaesthesis was not significantly correlated to typing ability in both groups.

In error detection higher typing ability coincided with less use of information from the keyboard in 10 finger typists. In idiosyncratic typists, higher typing ability coincided with more use of touch/kinaesthesis and more use of internal prediction.

The correlations between the number of fingers used by idiosyncratic typists and the extent of attention to/use of different sources of information are depicted in Table 5. The correlations show that participants who use more fingers pay less attention to the keyboard in free typing and copy typing. All other correlations were not significant.

**Table 5:** **Correlations between the number of fingers used by idiosyncratic typists and the extent of attention to/use of different sources of information during copy typing, free typing and error detection**.

	**Template**	**Screen**	**Keyboard**	**Fingers**	**Touch/kinaesthesis**	**Internal prediction**
Copy typing	0.001	0.14	−0.35^***^	−0.13	0.06	–
Free typing	–	0.11	−0.45^**^	−0.12	0.03	–
Error detection	–	0.12	−0.08	−0.03	0.14	0.14

** p < 0.01;

*** *p < 0.001*.

## Discussion

In the present study we investigated the extent of attention to different sources of information during free typing and copy typing and the use of different sources of information for error detection in 10 finger typists and idiosyncratic typists using self-reports. In addition, we analysed whether attention to some sources of information goes along with costs to other sources of information. Further, we were interested in the role of typing ability for the distribution of attention to different sources of information and whether the number of fingers used for typing in idiosyncratic typists is related to the distribution of attention.

### Attention in free typing and copy typing

In line with the expectations, 10 finger typists pay more attention to the template in copy typing, more attention to the screen in free typing, and less to movement related information (fingers and keyboard) in both typing tasks than idiosyncratic typists. These results reflect higher automaticity in processing movement related information in 10 finger typists. According to the two-loop theory of typewriting (Logan and Crump, 2009) the inner loop is responsible for generating keystrokes as well as monitoring the fingers and the keyboard in order to ensure that a typist moves the right fingers and hits the correct keys (Liu et al., 2010; Logan and Crump, 2011). Most likely the inner loop is more precise in 10 finger typists, which is reflected in automatic activation of keystrokes (Van den Bergh et al., 1990; Rieger, 2004; Beilock and Holt, 2007) and leaves 10 finger typists more attentional resources to attend to sources of information related to the outer loop. Idiosyncratic typists compensate for less precision in the inner loop by paying more attention to the fingers and the keyboard. This interpretation is corroborated by the correlations, which indicate that higher attention to sources of information related to the inner loop results in lower attention to sources of information related to the outer loop. Specifically, more attention to movement related information (fingers and keyboard) coincides with less attention to the screen in free typing in both groups, and less attention to the template in copy typing in 10 finger typists. Further, in idiosyncratic typists more attention to the fingers coincides with less attention to the screen in copy typing. These sources of information cannot be attended to at the same time, because typists have to look in different directions. Attention to the fingers and the keyboard were positively correlated, most likely because typists look into the same direction in order to acquire the respective information.

Unexpectedly, no significant differences in attention to touch/kinaesthesis between the groups were observed. We further observed that 10 finger typists' reported attention to touch/kinaesthesis did not significantly differ from their reported attention to the fingers (copy typing and free typing) and was not significantly different (copy typing) or even less (free typing) than attention to the keyboard. Thus, attention to touch/kinaesthesis was comparable to attention to sources of information to which 10 finger typists rarely attend. We had expected that 10 finger typists pay more attention to touch/kinaesthesis because tactile/kinaesthetic feedback is more specific in them than in idiosyncratic typists and should thus be of more use for 10 finger typists. Why did we not observe the expected effects? First, contrary to our assumption, touch/kinaesthesis may not be more important in 10 finger typists than in idiosyncratic typists and may not be as important as we thought. Nowadays 10 finger typists rarely adhere to the strict rules taught in classic touch typing lessons. However, results pointing to the importance of tactile/kinaesthetic feedback in skilled typing are not in accordance with this interpretation (Barrett and Krueger, 1994; Gordon and Soechting, 1995; Rabin and Gordon, 2004). Second, the question about touch/kinaesthesis may have been too unspecific. We assessed touch/kinaesthesis by asking to what extent participants pay attention to the feeling of moving their fingers. Maybe participants did not consider all aspects of touch/kinaesthesis in typing (e.g., how it feels to touch the key surfaces, the pressure on the skin when hitting a key, and the feeling of the movement of the fingers), resulting in an underestimation. A third explanation, which we think is the most likely one, is that even though tactile/kinaesthetic feedback is important in fast and automatic movements (Keele, 1968), participants might not be consciously aware of its importance during typing (at least as long as no errors occur, see below), because movement related information is automatically activated (Rieger, 2004; Beilock and Holt, 2007). This may even be functional, as well-learned skills like typing are believed to be based on automated control structures, largely outside explicit attentional control. Attention to such automatic processes even disturbs performance (Beilock and Carr, 2001; Logan and Crump, 2009).

Interestingly, we observed positive correlations between attention to the fingers and touch/kinaesthesis in idiosyncratic typists. It might be that participants had difficulties to distinguish between those two sources of information. However, the question referring to the fingers explicitly asked about looking at the fingers, whereas the question referring to touch/kinaesthesis asked about the feeling when moving the fingers. An alternative explanation is that vision of the fingers promotes tactile/kinaesthetic perception. Such cross-modal influences have previously been observed. For instance, compared to darkness or viewing neutral objects, viewing the own arm improves tactile discrimination at the arm (Kennett et al., 2001).

Copy typing requires attention to the template and therefore leaves less attentional capacity for other sources of information. Correspondingly, we observed that both 10 finger typists and idiosyncratic typists attend less to the screen and more to the template in copy typing. Interestingly, attention to the template and to the screen were negatively correlated in 10 finger typists but positively correlated in idiosyncratic typists. Most likely, in 10 finger typists a trade-off between attention to the screen and the template occurs, as both cannot bet attended to at the same time. Correspondingly, they report more attention to screen in free typing than in copy typing. In idiosyncratic typists, however, no trade-off occurs because the limiting factor for attention to the screen and the template may be the necessity to attend to movement related information (fingers and keyboard), which may be non-optional for many idiosyncratic typists. No significant difference between free typing and copy typing in attention to movement related information was observed, supporting this interpretation. However, the correlations only partly support this. Less attention to the screen was related to more attention to the fingers in copy typing, but attention to the template was not significantly correlated with attention to movement related information. No significant difference between free typing and copy typing was observed for touch/kinaesthesis in both idiosyncratic typists and 10 finger typists. Attention to touch/kinaesthesis may not be limited by attention to other sources of information like attention to the template. Thus, it may be attended to the same extent regardless of the typing task.

A further interesting effect was observed when free typing and copy typing were compared: 10 finger typists reported more attention to the keyboard in free typing than in copy typing. Most likely, this is a partly optional source of information which is attended to more when more attentional resources are available (i.e., attention to the template is not required). The result that 10 finger typists show more attention to the keyboard in free typing than in copy typing may seem surprising, because the keyboard is not an important source of information for them. However, even though taught not to do so, 10 finger typists do sometimes attend to the keyboard, for example to find uncommon keys (Long, 1976). Our results suggest that they do use the keyboard as a source of information in particular when their attention is not required elsewhere.

### Error detection

In both groups of typists visual feedback on the screen is used more often than any other source of information to detect errors. This corresponds to results showing that for the detection of some errors visual feedback is necessary (e.g., Long, 1976; Rabbitt, 1978; Hayes and Reeve, 1980) and that visual feedback facilitates error correction (Rieger et al., 2011) by providing information about the error type (West, 1967; Long, 1976). Further, typists preferentially rely on information from the screen, or, in terms of the two-loop theory of typing (Logan and Crump, 2009) on information from the outer loop, when they have to decide whether they actually made an error (Logan and Crump, 2011). They even report that they committed an error when they actually typed correctly, but an error is experimentally inserted, and they do not report an error when they committed one but this error is experimentally corrected (Logan and Crump, 2010). This is the case even though implicit error detection (measured by post error slowing, mediated by the inner loop) only occurs after actual errors but not after experimentally inserted errors (Logan and Crump, 2010; Snyder et al., 2015). Thus, other sources of information may contribute to error detection, but the final decision about the occurrence of an error largely depends on visual feedback from the screen.

In 10 finger typists the importance of the screen for error detection (they use it more often than any other source of information) was expected: their typing style facilitates more attention to the screen. Interestingly, however, 10 finger typists use the screen less than idiosyncratic typists to detect errors. This may seem surprising, as idiosyncratic typists report less attention to the screen than 10 finger typists in free typing. Idiosyncratic typists may rely more on the use of the screen for error detection, because other sources of information, like touch/kinaesthesis and internal prediction are less informative for them. In contrast, 10 finger typists use internal prediction and touch/kinaesthesis more, which may reduce the necessity to use the screen for error detection compared to idiosyncratic typists. Presumably, 10 finger typists have developed more precise internal models (Wolpert and Flanagan, 2001) of typing allowing them to detect errors based on a comparison between intended action consequences and internally predicted action consequences (cf. Rabbitt, 1978; Maidhof et al., 2009).

The observation, that touch/kinaesthesis is more informative in 10 finger typist than in idiosyncratic typists is consistent with previous studies (Gordon and Soechting, 1995; Rabin and Gordon, 2004; Crump and Logan, 2010). It might seem surprising that differences between the groups in the use of touch/kinaesthesis were observed in error detection, but not in free typing and copy typing. As argued above, touch/kinaesthesis might be a source of information, which is usually not consciously attended to during typing. However, errors disrupt the flow of intended action effects. A mismatch between intended and observed tactile/kinaesthetic feedback occurs and this mismatch might serve as a signal to become consciously aware of tactile/kinaesthetic feedback.

As expected, error detection based on attention to fingers and keyboard was more pronounced in idiosyncratic typists than in 10 finger typists. The use of the fingers and the use of the keyboard for error detection were positively correlated in both groups. This indicates that when a typist searches for the right key on the keyboard she or he also attends to the finger she or he wants to press it with. In particular during error detection, an error might be detected by comparing the intended and the actual position of the fingers on the keyboard.

### Typing ability

So far, we have discussed attention to different sources of information during typing depending on typing style. However users of a specific style may have higher or lower typing ability, which may also contribute to how attention is distributed during typing. We therefore correlated the extent of attention to different sources of information with self-reported typing ability and number of fingers used by idiosyncratic typists. In free typing and copy typing, we observed that participants with higher typing ability reported less attention to the fingers and the keyboard. In idiosyncratic typists, the use of more fingers coincided with less attention to the keyboard. Thus, proficiency in typing goes along with (implicit) knowledge about the location of the keys, regardless of typing style. In free typing, higher typing ability coincided with more attention to the screen (in idiosyncratic typists) and in copy typing higher typing ability coincided with more attention to the template (10 finger typists). Overall, idiosyncratic typists with higher typing ability partly distribute their attention in a way that is associated with 10 finger typing. Conversely, less proficient 10 finger typists partly distribute their attention in a way that is associated with idiosyncratic typing.

For error detection, higher typing ability coincided with less use of information from the keyboard in 10 finger typists. This is in accordance with the observation that higher typing ability coincides with less attention to the keyboard. In idiosyncratic typists higher typing ability coincided with more use of touch/kinaesthesis and internal prediction, supporting the assumption that higher skilled typists, regardless of typing style, rely more on sources of information which are usually associated with 10 finger typing.

Thus, proficiency in typing partly contributes to the distribution of attention during copy typing and free typing and to how errors are detected. However, even though proficiency is important, typing proficiency alone cannot explain all differences between typing styles. If this were the case, the patterns of correlations discussed above should be similar within each group. This was however not the case. For instance, in copy typing attention to the template and the screen were negatively correlated in 10 finger typists but positively correlated in idiosyncratic typists, indicating that different factors limit the trade-offs between attention to different sources of information. Thus, both proficiency and typing style contribute to how attention is distributed during typing.

### Limitations and perspectives

The use of self-reports is a limitation of the present study. Using self-reports to assess typing ability rather than an actual typing test might be subject to biases. For instance, estimating typing ability might depend on the chosen comparison group (i.e., does one type better than the average person he or she knows). However, the pattern of correlations in our study (e.g., higher self-reported typing ability is associated with less attention to movement related information) corresponds to studies using objective measurements of typing ability (Johansson et al., 2010). Thus, we think self- reported typing ability sufficiently reflects actual typing.

Further, using self-reports might have been problematic for assessing the distribution of attention. What participants think they are doing might not correspond to what they actually do or they may report what they think they should be doing (e.g., 10 finger typists might think they should not look at the keyboard and fingers). However, we think this is unlikely because not all results were what one would expect using common sense. For instance, 10 finger typists use the screen less than idiosyncratic typists to detect errors. Further, participants did not know that one aim of the study was to compare typists using different styles. Thus, we think it is likely that participants made an effort to report their actual typing behavior.

We think that our study provides useful data on the topic, because we had a large sample of participants and were thus able to investigate the influence of several factors on the distribution of attention to different sources of information during typing (typing style, typing skill, and number of fingers used in idiosyncratic typists). As sample sizes are usually much lower in experimental studies, some of these factors are usually kept constant and their influence is not investigated. Nevertheless, self-reports are just an initial step to approach this topic. In future studies eye movements might be recorded in different experimental conditions to observe actual behavior.

Models of typewriting usually deal with skilled 10 finger typing and do not take into account that people type using different styles. Further, the process of skill acquisition, and correspondingly typing proficiency, is also largely neglected. Thus, models might be extended to take those factors into account.

## Conclusion

In conclusion, our results indicate that typing task, typing proficiency, and typing style influence how attention is distributed during typing. In terms of the two-loop theory of typing (Logan and Crump, 2009, 2011; Liu et al., 2010), the inner loop is less finely tuned, or in terms of the internal models (Wolpert and Flanagan, 2001), internal models are less precise in idiosyncratic than in 10 finger typists. However, the present results also indicate that typing ability contributes to the distribution of attention during typing. Regardless of typing style higher skilled typists rely more on the sources of information which are usually associated with 10 finger typing and lower skilled typist rely more on the sources of information which are usually associated with idiosyncratic typing. Thus, in addition to typing style *per-se*, proficiency within the own style plays an important role for how attention is distributed and how errors are detected.

## Ethics statement

The study was approved by the Research Committee for Scientific and Ethical Questions (RCSEQ). All participants gave written informed consent.

## Author contributions

MR contributed to designing the research, analyzing the data and writing the manuscript. VB contributed to collecting and analyzing the data and writing the manuscript.

### Conflict of interest statement

The authors declare that the research was conducted in the absence of any commercial or financial relationships that could be construed as a potential conflict of interest.

## References

[B1] Barrett J. Krueger H. (1994). Performance effects of reduced proprioceptive feedback on touch typists and casual users in a typing task. Behav. Inf. Technol. 13, 373–381. 10.1080/01449299408914618

[B2] Beilock S. L. Carr T. H. (2001). On the fragility of skilled performance: what governs choking under pressure? J. Exp. Psychol. Gen. 130, 701–725. 10.1037/0096-3445.130.4.701 11757876

[B3] Beilock S. L. Holt L. E. (2007). Embodied preference judgments: Can likeability be driven by the motor system? Psychol. Sci. 18, 51–57. 10.1111/j.1467-9280.2007.01848.x 17362378

[B4] Cooper W. E. (1983). Introduction, in Cognitive Aspects of Skilled Typewriting, ed Cooper W. E. (New York, NY: Springer Verlag), 1–38.

[B5] Crump M. J. C. Logan G. D. (2010). Warning: this keyboard will deconstruct–the role of the keyboard in skilled typewriting. Psychon. Bull. Rev. 17, 394–399. 10.3758/PBR.17.3.394 20551364

[B6] Diehl M. J. Seibel R. (1962). The relative importance of visual and auditory feedback in speed typewriting. J. Appl. Psychol. 46, 365–369. 10.1037/h0041438

[B7] Gordon A. M. Soechting J. F. (1995). Use of tactile afferent information in sequential finger movements. Exp. Brain Res. 107, 281–292. 10.1007/BF00230048 8773246

[B8] Hayes V. Reeve T. G. (1980). Role of visual feedback for response guidance and response confirmation in typewriting. Percept. Mot. Skills 50, 1047–1056. 10.2466/pms.1980.50.3c.1047

[B9] Johansson R. Wengelin A. Johansson V. Holmqvist K. (2010). Looking at the keyboard or the monitor: relationship with text production processes. Read. Writ. 23, 835–851. 10.1007/s11145-009-9189-3

[B10] Jordan M. I. (1995). The organization of action sequences: evidence from a relearning task. J. Motor Behav. 27, 179–192. 10.1080/00222895.1995.9941709 12736126

[B11] Keele S. W. (1968). Movement control in skilled motor performance. Psychol. Bull. 70, 387–403. 10.1037/h0026739

[B12] Kennett S. Taylor-Clarke M. Haggard P. (2001). Noninformative vision improves the spatial resolution of touch in humans. Curr. Biol. 11, 1188–1191. 10.1016/S0960-9822(01)00327-X 11516950

[B13] Liu X. Crump M. J. Logan G. D. (2010). Do you know where your fingers have been? Explicit knowledge of the spatial layout of the keyboard in skilled typists. Memory Cogn. 38, 474–484. 10.3758/MC.38.4.474 20516227

[B14] Loeb K. M. C. (1983). Membrane keyboards and human performance. Bell Syst. Tech. J. 62, 1733–1749. 10.1002/j.1538-7305.1983.tb03511.x

[B15] Logan G. D. Crump M. J. (2009). The left hand doesn't know what the right hand is doing: the disruptive effects of attention to the hands in skilled typewriting. Psychol. Sci. 20, 1296–1300. 10.1111/j.1467-9280.2009.02442.x 19765236

[B16] Logan G. D. Crump M. J. C. (2010). Cognitive illusions of authorship reveal hierarchical error detection in skilled typists. Science 330, 683–686. 10.1126/science.1190483 21030660

[B17] Logan G. D. Crump M. J. (2011). Hierarchical control of cognitive processes: the case for skilled typewriting, in The Psychology of Learning and Motivation, ed Ross B. (Burlington, VT: Academic Press), 1–27.

[B18] Long J. (1976). Visual Feedback and skilled keying: differential effects of masking the printed copy and the keyboard. Ergonomics 19, 93–110. 10.1080/00140137608931517 26528599

[B19] Long J. Nimmo-Smith I. Whitefield A. (1983). Skilled typing: a characterization based on the distribution of times between responses, in Cognitive Aspects of Skilled Typewriting, ed Cooper W. E. (New York, NY: Springer Verlag), 145–195.

[B20] Maidhof C. Rieger M. Prinz W. Koelsch S. (2009). Nobody is perfect: ERP effects prior to performance errors in musicians indicate fast monitoring processes. PLoS ONE 4:e5032. 10.1371/journal.pone.0005032 19337379 PMC2660409

[B21] Rabbitt P. (1978). Detection of errors by skilled typists. Ergonomics 21, 945–958. 10.1080/00140137808931800

[B22] Rabin E. Gordon A. M. (2004). Tactile feedback contributes to consistency of finger movements during typing. Exp. Brain Res. 155, 362–369. 10.1007/s00221-003-1736-6 14689143

[B23] Rieger M. (2004). Automatic keypress activation in skilled typing. J. Exp. Psychol. Hum. Percept. Perform. 30, 555–565. 10.1037/0096-1523.30.3.555 15161386

[B24] Rieger M. (2007). Letters as visual action-effects in skilled typing. Acta Psychol. (Amst) 126, 138–153. 10.1016/j.actpsy.2006.11.006 17250793

[B25] Rieger M. (2012). Motor imagery in typing: effects of typing style and action familiarity. Psychon. Bull. Rev. 19, 101–107. 10.3758/s13423-011-0178-6 22057418

[B26] Rieger M. Martinez F. Wenke D. (2011). Imagery of errors in typing. Cognition 121, 163–175. 10.1016/j.cognition.2011.07.005 21821234

[B27] Roeber H. Bacus J. Tomasi C. (2003). Typing in thin air the canesta projection keyboard – a new method of interaction with electronic devices, in Proceedings of the ACM CHI 2003 Human Factors in Computing Systems Conference, eds Cockton G. Korhonen P. (Ft. Lauderdale, FL: ACM Press), 712–713.

[B28] Snyder K. M. Logan G. D. Yamaguchi M. (2015). Watch what you type: The role of visual feedback from the screen and hands in skilled typewriting. Atten. Percept. Psychophys. 77, 282–292. 10.3758/s13414-014-0756-6 25142895

[B29] Tapp K. M. Logan G. D. (2011). Attention to the hands disrupts skilled typewriting: The role of vision in producing the disruption. Atten. Percept. Psychophys. 73, 2379–2383. 10.3758/s13414-011-0208-5 21915764

[B30] Van den Bergh O. Vrana S. Eelen P. (1990). Letters from the heart: affective categorization of letter combinations in typists and nontypists. J. Exp. Psychol. Learn. Memory Cogn. 16, 1153–1161. 10.1037/0278-7393.16.6.1153

[B31] West L. J. (1967). Vision and kinesthesis in the acqusition of typewriting skill. J. Appl. Psychol. 51, 161–166. 10.1037/h0024325 6039340

[B32] West L. J. (1983). Acquisition of Typewriting Skills. Indianapolis, IN: Bobbs-Merrill Educational Publishing.

[B33] Wolpert D. M. Flanagan J. R. (2001). Motor prediction. Curr. Biol. 11, 729–732. 10.1016/S0960-9822(01)00432-8 11566114

