# Supplementary material for: Typing Style and the Use of Different Sources of Information during Typing: An Investigation Using Self-Reports
Source: Front Psychol. 2016 Dec 9;7:1908. doi: 10.3389/fpsyg.2016.01908 (PMC5145878; doi:10.3389/fpsyg.2016.01908)
Supplement: Supplementary file 1 [file DataSheet1.pdf]

SUPPLEMENTAL MATERIAL

Typing Style and the Use of Different Sources of Information during Typing: An Investigation Using Self-reports

Martina Rieger & Victoria K. E. Bart[1]

1 UMIT - University for Health Sciences, Medical Informatics and Technology

A. Questions on typing ability

A.1 Original questions, in German

Wie viele Stunden in der Woche verbringen Sie durchschnittlich damit auf einer Tastatur zu Tippen?

Seit wie vielen Jahren schreiben Sie auf einer Tastatur?

Wie schätzen Sie Ihre Fähigkeit zu Tippen ein?
sehr schlecht |--| sehr gut

A.2 English translation

How many hours a week on average do you spend typing on a keyboard?

How many years have you been typing on a keyboard?

How do you rate your ability to type?
very bad |--| very good

B. Questions on attention to different sources of information in typing

B.1 Original questions, in German

Bitte überlegen Sie, in welchem Ausmaß ihre Aufmerksamkeit/ihr Blick beim **FREIEN TIPPEN** (z. B. wenn Sie eigene Gedanken aufschreiben) auf verschiedene Aspekte des Tippens gerichtet ist.

Ich schaue auf meine Finger:
nie selten manchmal oft immer

Ich schaue auf die Tastatur selbst:
nie selten manchmal oft immer

Ich schaue auf den Bildschirm am Computer:
nie selten manchmal oft immer

Ich beachte wie es sich anfühlt, meine Finger zu bewegen:
nie selten manchmal oft immer

Bitte überlegen Sie, in welchem Ausmaß ihre Aufmerksamkeit/ihr Blick beim **ABTIPPEN** von einer Vorlage auf verschiedene Aspekte des Tippens gerichtet ist.

Ich schaue auf die Vorlage:
nie selten manchmal oft immer

Ich schaue auf meine Finger:
nie selten manchmal oft immer

Ich schaue auf die Tastatur selbst:
nie selten manchmal oft immer

Ich schaue auf den Bildschirm am Computer:
nie selten manchmal oft immer

Ich beachte wie es sich anfühlt, meine Finger zu bewegen:
nie selten manchmal oft immer

Wie bemerken Sie **TIPPFEHLER**?

Einen Tippfehler bemerke ich dadurch, dass am Bildschirm ein Fehler aufgetreten ist:
nie selten manchmal oft immer

Einen Tippfehler bemerke ich dadurch, dass ich auf meine Finger schaue:
nie selten manchmal oft immer

Einen Tippfehler bemerke ich dadurch, dass ich auf die Tastatur schaue:
nie selten manchmal oft immer

Einen Tippfehler bemerke ich dadurch, dass sich meine Fingerbewegungen komisch anfühlen:
nie selten manchmal oft immer

Einen Tippfehler bemerke ich auch wenn ich weder auf den Bildschirm, noch auf die Tastatur, noch auf meine Finger schaue, und sich auch die Finger nicht komisch anfühlen – ich weiß auch ohne diese Informationen, dass jetzt gleich etwas schieflaufen wird:
nie selten manchmal oft immer

B.2 English translation

Please indicate to which extent you pay attention to/look at different aspects of typing during **free typing** (e.g. when you write down your own thoughts).

I look at my fingers:
never rarely sometimes often always

I look at the keyboard:
never rarely sometimes often always

I look at the screen:
never rarely sometimes often always

I pay attention to what it feels like to move my fingers:
never rarely sometimes often always

Please indicate to which extent you pay attention to/look at different aspects of typing during **copy typing.**

I look at the template:
never rarely sometimes often always

I look at my fingers:
never rarely sometimes often always

I look at the keyboard:
never rarely sometimes often always

I look at the screen:
never rarely sometimes often always

I pay attention to what it feels like to move my fingers:
never rarely sometimes often always

How do you **detect typing errors?**

I detect a typing error by looking at the screen:
never rarely sometimes often always

I detect a typing error by looking at my fingers:
never rarely sometimes often always

I detect a typing error by looking at the keyboard:
never rarely sometimes often always

I detect a typing error because my finger movements feel weird:
never rarely sometimes often always

I detect a typing error even if I do not look at the screen, the keyboard or the fingers, and my finger movements do not feel weird. I know without this information, that something is about to go wrong:
never rarely sometimes often always
